# FEM-Based Design of Mass–Spring Acoustic Matching Layers for Ultra-High-Frequency Ultrasonic Transducers with Half-Concave Piezoelectric Elements

**DOI:** 10.3390/s26144334

**Published:** 2026-07-08

**Authors:** Jianxin Zhao, Zhipeng Zhang, Zhaoxi Li, Yugao Li, Yaocheng Li, Guohua Huang, Yintang Yang

**Affiliations:** 1 Northwest Institute of Mechanical and Electrical Engineering, Xianyang 712099, China; 15002949671@163.com (J.Z.); 19909450127@163.com (Y.L.); lizi2033@163.com (Y.L.); hgh1999@foxmail.com (G.H.); 2 Faculty of Integrated Circuits, Xidian University, Xi’an 710071, China; lizhaoxi@xidian.edu.cn (Z.L.); ytyang@xidian.edu.cn (Y.Y.)

**Keywords:** ultrasonic transducer, ultra-high frequency, mass–spring, acoustic matching layer, half-concave

## Abstract

High-frequency ultrasonic transducers are pivotal for detecting minute defects, offering distinct advantages in terms of non-destructive evaluation, non-invasiveness, and superior spatial resolution. However, achieving effective focusing and efficient acoustic transmission for ultra-high-frequency ultrasonic transducers is a significant challenge. To address this challenge, a mass–spring acoustic matching layer is designed for a transducer based on a half-concave LiNbO_3_ piezoelectric element at 100 MHz. The proposed mass–spring stack, comprising a 0.25 μm Au layer and a 2.5 μm Parylene-C layer, operates within a deposition-friendly thickness range ideal for curved substrates, while a conventional quarter-wavelength Parylene-C layer would necessitate a thickness of 6.0 μm at this frequency. The transducer is modeled in COMSOL Multiphysics 6.1, coupling solid mechanics, electrostatics with piezoelectric effects, and pressure acoustics for the water load. Using a fixed concave geometry (curvature radius is 1 mm, which means the focal length is also 1 mm), a frequency sweep from 50 MHz to 150 MHz is conducted to evaluate performance. Analysis of the pressure distribution in the focal plane reveals that the focal length and the −6 dB beamwidth are predominantly governed by aperture diffraction and exhibit minimal variation upon incorporation of a matching layer. The focal length is approximately 1.02 mm within the excitation frequency range of 70 MHz–150 MHz, and the beamwidth decreases markedly with increasing frequency. At 100 MHz, the measured −6 dB beamwidth is approximately 31.6 μm without a matching layer, 32.3 μm with a quarter-wavelength matching layer, and 31.0 μm with a mass–spring matching layer. Crucially, quantitative comparisons reveal that this design yields a systematically higher focal pressure; near the 100 MHz design frequency, the acoustic pressure amplitude achieved with the mass–spring configuration is 1.5 times greater than that obtained using the quarter-wavelength reference. These research results provide a theoretical basis for the application of ultra-high-frequency ultrasound in the detection of tiny defects.

## 1. Introduction

Ultrasonic transducers are widely used in the non-destructive testing of high-precision equipment, especially in the detection of defects in flip-chip bonding [1,2,3,4,5]. Ultrasound, as a physical wave modality, is intrinsically non-invasive and non-destructive, while what is distinctive at ≥100 MHz is the wavelength scaling (λ ≈ 15 µm in water) that enables micron-scale spatial resolution and fine-feature interrogation. However, as the frequency continues to increase, especially in the ultra-high-frequency (>100 MHz) stages, how to achieve effective focusing and efficient acoustic transmission efficiency of ultra-high-frequency ultrasonic transducers has been the goal that researchers have strived toward.

The traditional focusing methods for high-frequency (>20 MHz) ultrasonic transducers include mechanical pressure focusing and lens focusing. The mechanical pressure focusing method involves applying mechanical pressure to the originally planar piezoelectric element, causing it to undergo elastic deformation and form a temporary spherical shape, thereby changing the directionality of the acoustic beam. This method is highly flexible, but its stability may be limited [6,7,8]. Lens focusing is a focusing technique that achieves acoustic beam convergence through the refraction effect of an externally added spherical acoustic lens. The core of this method is to utilize the difference in sound velocity between the lens material and the surrounding medium, causing the ultrasonic wave to converge towards the focus after being refracted by the spherical surface. However, the lens material will absorb/reflect a portion of the acoustic energy (especially at high frequencies), resulting in a decrease in transmission efficiency [9,10,11]. The half-concave self-focusing structure is a self-focusing technology that achieves acoustic beam convergence by designing the radiation surface of the transducer to be a partially concave spherical surface. It does not require an external lens or mechanical pressure for focusing [5,12,13]. While maintaining the focusing function, it can reduce the overall size, adapt to compact spaces, and the fabricated transducer has significantly improved stability and sensitivity.

To enhance the acoustic transmission efficiency of ultrasonic transducers, researchers often employ the quarter-wavelength principle to design acoustic matching layers [14,15,16]. These layers utilize materials with specific thicknesses and impedances to cancel out the reflected waves at the interface. Due to the limitations of material selection and fabrication process, the quarter-wavelength acoustic matching method cannot be applied to the half-concave high-frequency ultrasonic transducer. The proposal of the mass–spring (M-S) method has broken this deadlock [17,18]. It overcomes the limitations of the traditional quarter-wavelength matching method at high frequencies through mechanical vibration analogy. This method regards the high impedance layer (Au) as a “mass block” and the low-impedance layer (Parylene C) as a “spring”, forming a resonance system, thereby achieving continuous adjustment of acoustic impedance without relying on the specific material impedance.

This study designs an ultra-high-frequency ultrasonic transducer based on half-concave piezoelectric elements, where the highest center frequency is precisely controlled by the thickness of the thinnest area of the element. By innovatively adopting an uneven thickness structure, multiple vibration modes are induced to couple cooperatively, significantly expanding the effective working bandwidth and breaking through the frequency limitations of traditional uniform thickness transducers. To address the issue of acoustic impedance mismatch, a novel acoustic matching layer based on the mass–spring model is proposed. By equivalently simplifying the acoustic impedance matching mechanism, the energy transmission path is optimized. Simulation results show that after loading this matching layer, the acoustic pressure amplitude at the focus area of the transducer is significantly enhanced, and the acoustic energy transmission efficiency is increased by more than 40% compared to the structure without the matching layer, verifying its excellent acoustic performance in the ultra-high-frequency range. The half-concave structure combined with geometric focusing characteristics further enhances the beam convergence ability, reducing the focal spot diameter to sub-millimeter level. These research results lay an important theoretical basis for the application of ultra-high-frequency ultrasound in the detection of tiny defects, and provide a new idea for the broadband design of high-performance ultrasonic transducers.

## 2. Design of Half-Concave Piezoelectric Elements and Mass–Spring Acoustic Matching Layers

### 2.1. Half-Concave Piezoelectric Element

**Figure 1:**
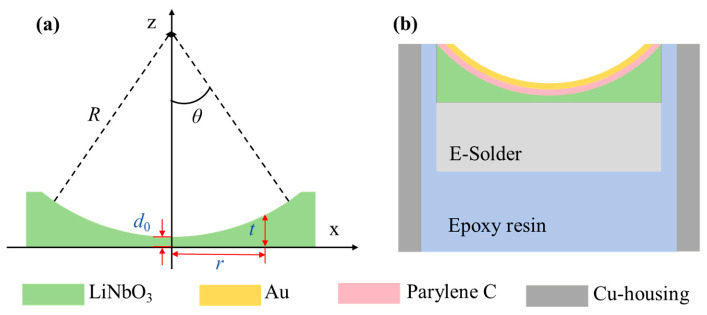
Schematic diagrams: (**a**) half-concave piezoelectric element, (**b**) ultrasonic transducer.

As illustrated in Figure 1a, a half-concave configuration was adopted. The piezoelectric element, fabricated from a single-crystal material, features a smoothly contoured concave aperture defined by a half-circular arc. Through the piezoelectric effect, it achieves efficient excitation and regulation of acoustic waves. This structure utilizes the geometric characteristics of the concave surface to enhance the focusing ability of the acoustic field. Combined with the inverse piezoelectric effect of the piezoelectric material, it generates directional mechanical vibration under the excitation of an electrical signal and radiates high-directional acoustic waves. The function *t*, derived from the schematic in Figure 1b, is given as follows [19,20]:

(1) $$t=R+d_{0}-\sqrt{R^{2}-r^{2}}$$

where *d*_0_ represents the thinnest part of the element, *R* indicates the radius of curvature, and r represents the distance from a certain position within the piezoelectric element to the center. In this study, the active LiNbO_3_ face is finished as a spherical cap, with a radius of curvature *R* = 1 mm, an aperture radius *a* = 0.5 mm, and the center frequency of ultrasonic transducer is about 100 MHz.

### 2.2. Mass–Spring Acoustic Matching Layers

A classical single λ/4 layer requires a predetermined acoustic impedance $Z_{1}=\sqrt{Z_{P}^{2}Z_{l}}$ (*Z*_p_ and *Z*_l_ represent the acoustic impedances of the piezoelectric material and the front-end load, respectively) and a thickness locked at $t=\lambda_{m}/4=v_{m}/4f_{0}$ (λ_m_, *v*_m_, and *f*_0_ represent the wavelength, the acoustic velocity of the matching layer, and the center frequency, respectively). For typical metal-particle/polymer composites, *v*_m_ ≈ 1800–2000 m/s, so at 100 MHz, the λ/4 thickness is only about 5 µm. At this scale, the layer thickness becomes comparable to—or smaller than—common conductive/ceramic filler sizes (>1 µm), and the composite ceases to behave as an effectively homogeneous continuum; the local impedance fluctuates and scattering/roughness losses increase, all while achieving and certifying that a uniform sub µm thickness tolerance across a curved aperture by lapping/spinning is intrinsically fragile. Moreover, with a curved face, any planar lap plate contacts only locally; thickness inevitably becomes a function of the azimuth/polar position, producing an aperture-dependent resonance and phase error (focal degradation/pulse distortion) that originates from the realization constraints rather than from the λ/4 principle itself.

The matching strategy adopted in this study does not rely on realizing a homogeneous, particle-filled composite layer at a prescribed quarter-wavelength thickness; instead, it employs a deposition-defined metal–polymer stack in which the “mass” and “spring” functions are provided by an ultra-thin sputter-deposited metallization (Cr/Au) and a conformal poly (Parylene C) film introduced by chemical vapor deposition (CVD). Crucially, parylene CVD proceeds via gas-phase transport and surface adsorption/polymerization under conditions where the molecular mean free path is comparable to relevant geometric length scales; thus, the flux effectively illuminates all exposed surfaces regardless of local viewing angle. Au provides a dense, low-loss conductor that can be deposited as a rigid, sub-micron skin whose effective role is that of a non-deforming mass, while Parylene C acts as a light, compliant polymer whose thickness can still be set by vapor deposition rather than by lapping a particle-filled composite; the pair therefore creates a very large impedance contrast on the order of twenty-five times, which satisfies the lumped-element premise that the high-impedance layer carries the inertia and the low-impedance layer supplies the spring constant—it also keeps the whole stack conformal on a curved surface.

**Figure 2:**
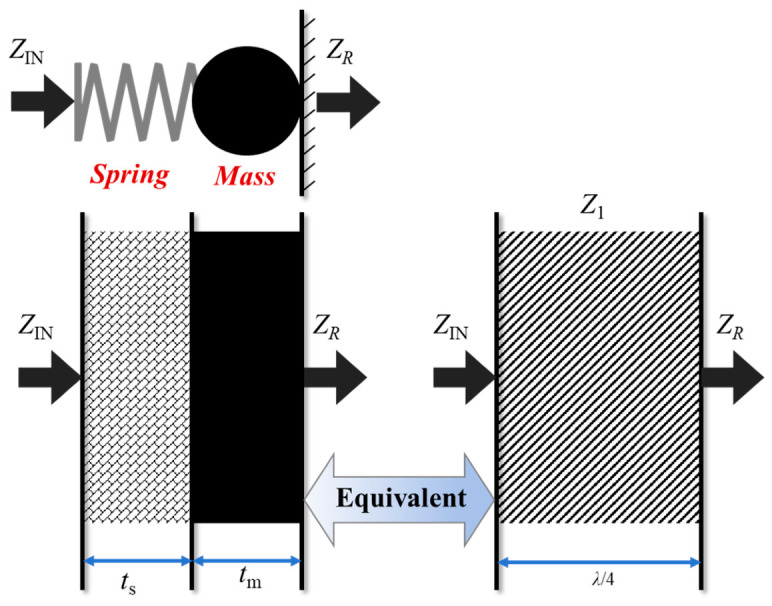
Schematic diagram of mass–spring acoustic matching layers.

The mass–spring structure in this study is an acoustic system design based on the principle of mechanical resonance. Figure 2 shows the schematic diagram of mass–spring acoustic matching layer. It comprises a mass block (mass) and an elastic support (spring), which aim to optimize the transmission efficiency of high-frequency acoustic waves through impedance matching. The mass comprises the base material (metal with a density of *ρ*_m_ and thickness of *t*_m_) and the spring material (with a density of *ρ*_s_ and thickness of *t*_s_), and the total mass–spring system is given as follows [21,22]:

(2) $$M=\rho_{m}t_{m}+0.4\rho_{s}t_{s}$$

where 0.4 represents the effective mass coefficient of the piezoelectric material. The mass cooperates with the spring through inertial effect, determining the resonant frequency *ω* of the system to be

(3) $$\omega =\sqrt{K/M}$$

The spring provides a restoring force *K*, maintaining the mechanical resonance characteristics of the system. When the system resonates, in order to ensure efficient storage and release of energy, the following conditions (Equation (4)) need to be met.

(4) $$-\omega^{2}M+K=0$$

By applying the transmission line theory and adjusting the structure length *l* to make the wavenumber *βl* = π/2, the input impedance *Z*_IN_ is transformed into a function of the dielectric characteristic impedance *Z*_m_:

(5) $$Z_{IN}=\frac{\rho_{s}{v_{s}}^{2}\left(\rho_{m}t_{m}+0.4\rho_{s}t_{s}\right)}{t_{s}Z_{R}}$$

where *Z*_R_ represents the radiation impedance, and *v*_p_ is the sound velocity of the piezoelectric material. The resonant frequency *ω* of the system is calculated as:

(6) $$\omega =\sqrt{K/M}=\sqrt{\frac{\rho_{s}{v_{s}}^{2}/t_{s}}{\rho_{m}t_{m}+0.4\rho_{s}t_{s}}}$$

The mass–spring model achieves the equivalent of the quarter-wavelength acoustic impedance matching method through the synergy of the mass and spring, enabling efficient focusing and energy transmission of high-frequency ultrasound.

### 2.3. Finite Element Simulation Model

**Figure 3:**
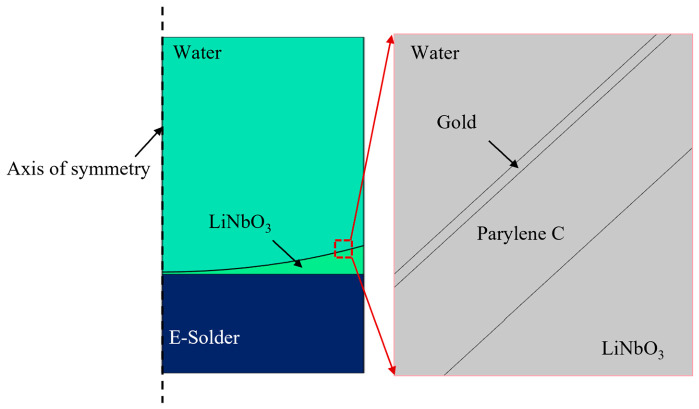
Finite element model for acoustic field simulation.

As shown in Figure 3, to validate the focusing performance of the half-concave piezoelectric element, a two-dimensional (2D) axisymmetric finite element model was constructed based on a finite element software, and simulation analysis was conducted. The employed lithium niobate (LiNbO_3_) material parameters were: density (*ρ*) of 4688 kg/m^3^; sound speed (*v*) of 7360 m/s; and piezoelectric coefficient *d*_33_ = 35 pC/N, whose excellent piezoelectric properties qualify it for the fabrication of high-frequency transducers. A highly attenuative conductive epoxy resin (E-solder 3022) was selected as the backing layer to reduce acoustic wave reflection from the rear of the piezoelectric element. For the matching layer, Au (density: 19,700 kg/m^3^; sound velocity: 3240 m/s) and Parylene C (*ρ* = 1100 kg/m^3^, *v* = 2350 m/s) were chosen. Table 1 lists the material parameters used in the finite element simulation [18]. During the simulation, pressure acoustic, solid mechanic, and electrostatic physics fields were selected to accurately characterize acoustic wave propagation. Among them, Au, Parylene C, and water were assigned to the pressure acoustic field, while LiNbO_3_ was associated with the solid mechanic and electrostatic fields. To simulate an infinite water environment and eliminate interference from non-experimental factors, a perfectly matched layer (PML) was applied to the outermost layer of the model. Finally, the mesh was constructed to resolve the disparate physics and geometric scales present in the ultra-high-frequency piezoelectric model. Given the 100 MHz operational frequency and the sub-micron thickness of the matching layers, a physics-controlled mesh was configured with extremely fine settings applied to the solid domains, while the fluid domain used a fine setting to capture the acoustic wavelength in water. The minimum element size in this study was set to approximately one-fifth of the smallest wavelength or layer thickness to ensure numerical convergence. The meshing method used was “Mapped”. The most critical regions (piezoelectric element and mass–spring stack) were subjected to boundary layer meshing (number of units in the “Distribution” is set to 5 in this study) to accurately resolve the steep gradients in stress and the electric field near the curved surface. The excitation frequency sweep range was set to 50 MHz–150 MHz to obtain acoustic field distributions at different frequencies.

In this study, using this commercial software, the acoustic field distribution of a high-frequency ultrasonic transducer was simulated. Specifically, a 2D axisymmetric model of a half-concave piezoelectric element with a diameter of 1 mm was constructed. The acoustic impedance of the piezoelectric material LiNbO_3_ was 34.5 MRayl, while the load was water with an acoustic impedance of 1.5 MRayl. Therefore, the acoustic impedance *Z*_1_ of the matching layers can be calculated by

(7) $$Z_{1}=\sqrt[{3}]{Z_{IN}^{2}Z_{R}}$$

In this specific case and in association with the selected materials (Table 1), an equivalent acoustic impedance *Z*_1_ was 12.13 MRayl. In this study, the center frequency of the ultrasonic transducer was 100 MHz; that is, f = 100 MHz. The mass–spring method uses the high- and low-impedance layers of a mass and spring to form a resonator system. By defining the densities of the spring layer and mass layer materials, the approximate resonance frequency (f=2π/ω) of a two-layer mass–spring combination, as well as the equivalent impedance at the driven end of spring *Z*_1_ (boundary with the piezoelectric layer), can be derived using Equations (6) and (7). Substituting the material parameters in Table 1 into Equation (5) will yield *t*_m_ = 0.1337 *t*_s_. By substituting these results into Equation (5), we can obtain *t*_s_ = 2.377 μm and *t*_m_ = 0.271 μm. Through further simplification, based on the results of Reference [18], the approximate result is taken as follows: Au, 0.25 μm; and Parylene C, 2.5 μm. In this study, the minimum thickness of the half-concave piezoelectric layer is 10 μm, and the thickness of E-solder 3022 is 500 μm.

## 3. Results

This study focuses on the simulation of the focused acoustic field of a 100 MHz LiNbO_3_ ultrasonic transducer (with a Au–Parylene C composite layer as the matching layer) design based on the mass–spring method. The COMSOL Multiphysics software was used in an aqueous medium (water) environment, with a particular emphasis on analyzing the characteristics of the focus, energy distribution, and resolution performance.

**Figure 4:**
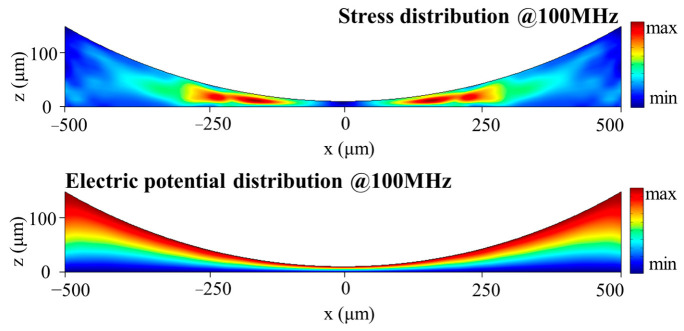
Simulation stress and electric potential distribution diagrams of a half-concave piezoelectric element.

As shown in Figure 4, at an excitation frequency of 100 MHz, the simulated stress distribution reveals that the maximum stress occurs at a radial distance of approximately 200 μm from the center of the half-concave piezoelectric element. In contrast, the electric potential distribution is essentially uniform from the concave surface to the bottom of the element, indicating that the electrostatic field within the piezoelectric volume is dominated by the terminal boundary condition and is not perturbed by the curved geometry to the same degree as the mechanical stress field.

**Figure 5:**
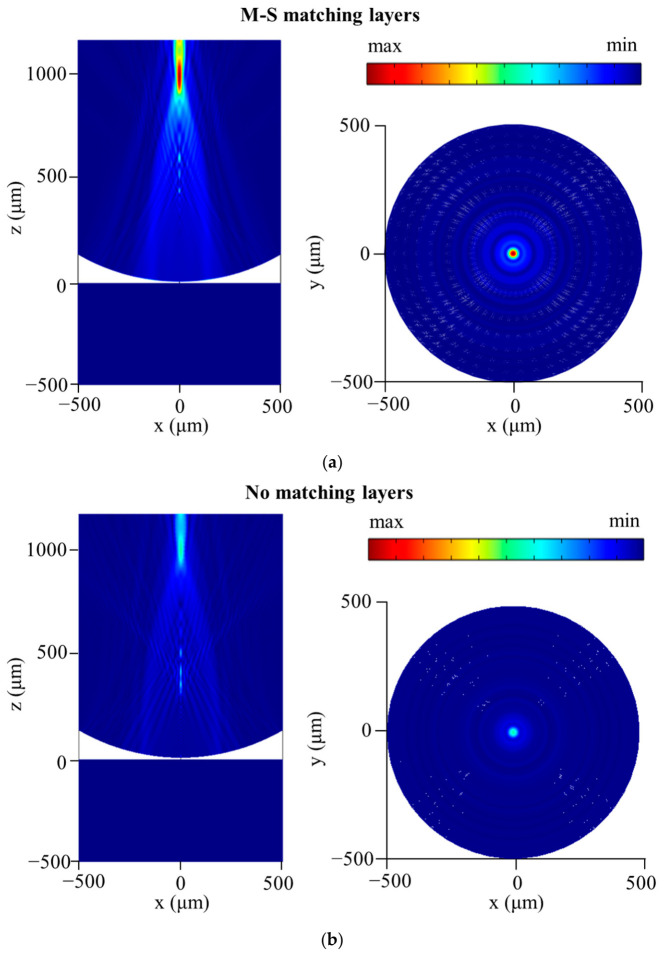
Normalized acoustic field distribution at 100 MHz: (**a**) no matching layer, (**b**) mass–spring acoustic matching layers. The acoustic field distribution map is normalized to the maximum value of the acoustic pressure amplitude formed by the ultrasonic transducers without matching layers and with the M-S matching layers.

The acoustic field simulation results show that the optimized transducer achieves a clear Gaussian-type-focused acoustic field, as shown in Figure 5a,b, at a 100 MHz operating frequency; as such, the focus is located at the designed focal distance (1 mm), the acoustic pressure distribution exhibits significant spatial localization characteristics, and the acoustic pressure amplitude at the center of the focus is approximately 1.3 times higher than that of the design without the matching layer. The focal region (the area with half the width of the acoustic pressure) is ellipsoidal. Notably, the acoustic field distribution map is normalized to the maximum value of the acoustic pressure amplitude formed by the ultrasonic transducers. 

**Figure 6:**
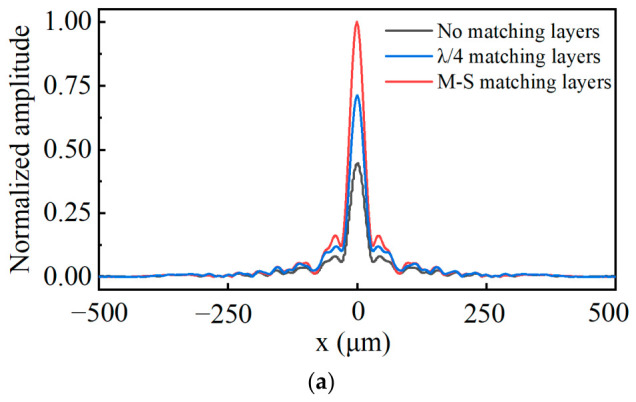

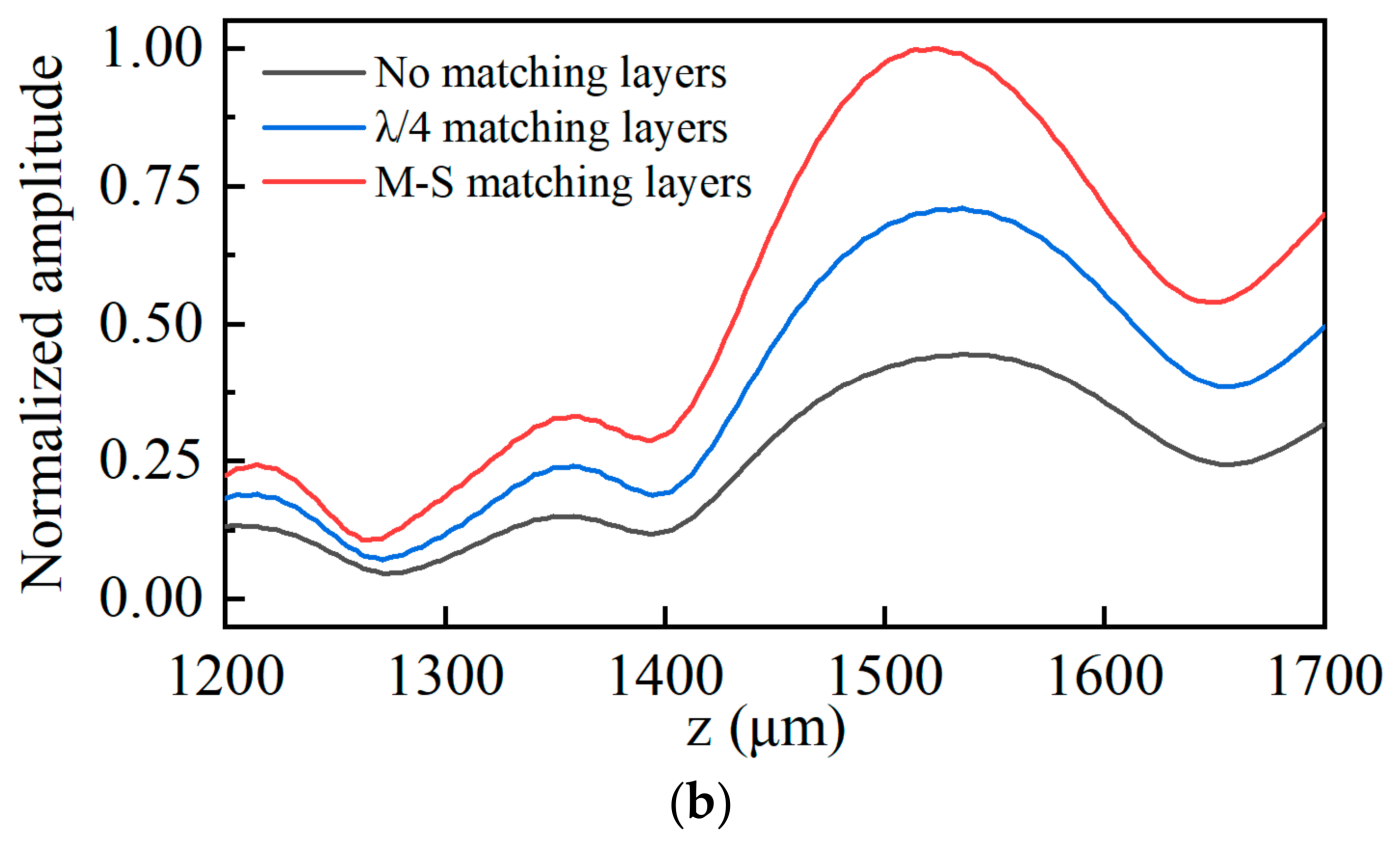
Normalized acoustic pressure amplitude distribution at the focal plane: (**a**) lateral axis, (**b**) axial axis.

In addition, we conducted comparative simulations and analyses using Parylene C as the quarter-wavelength matching layer material. For a homogeneous single matching layer, the quarter-wavelength condition gives the familiar relation $t=\lambda_{m}/4=v_{m}/4f_{0}$. At the center frequency *f*_0_ = 100 MHz, it can be calculated that the thickness of the Parylene-C is 6 μm. The spatial resolution curve, as shown in Figure 6, was obtained through statistics. The lateral (x-y plane) half full width at half maximum is 32 μm, corresponding to a theoretical horizontal resolution of approximately 25 μm. This deviation might be caused by the uneven thickness of the piezoelectric element, resulting in aberration. The transducer’s lateral resolution is excellent, meeting the requirements of high-frequency microscopic imaging.

**Figure 7:**
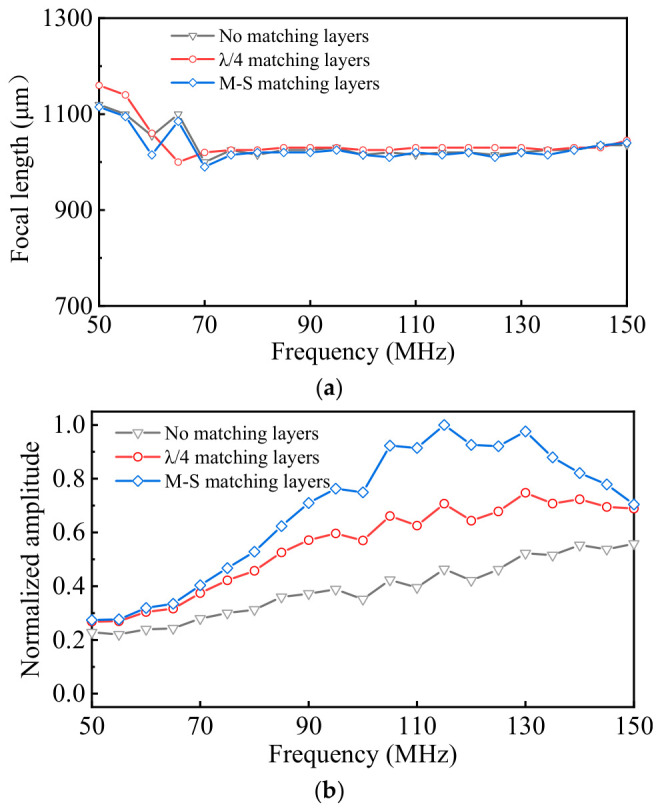

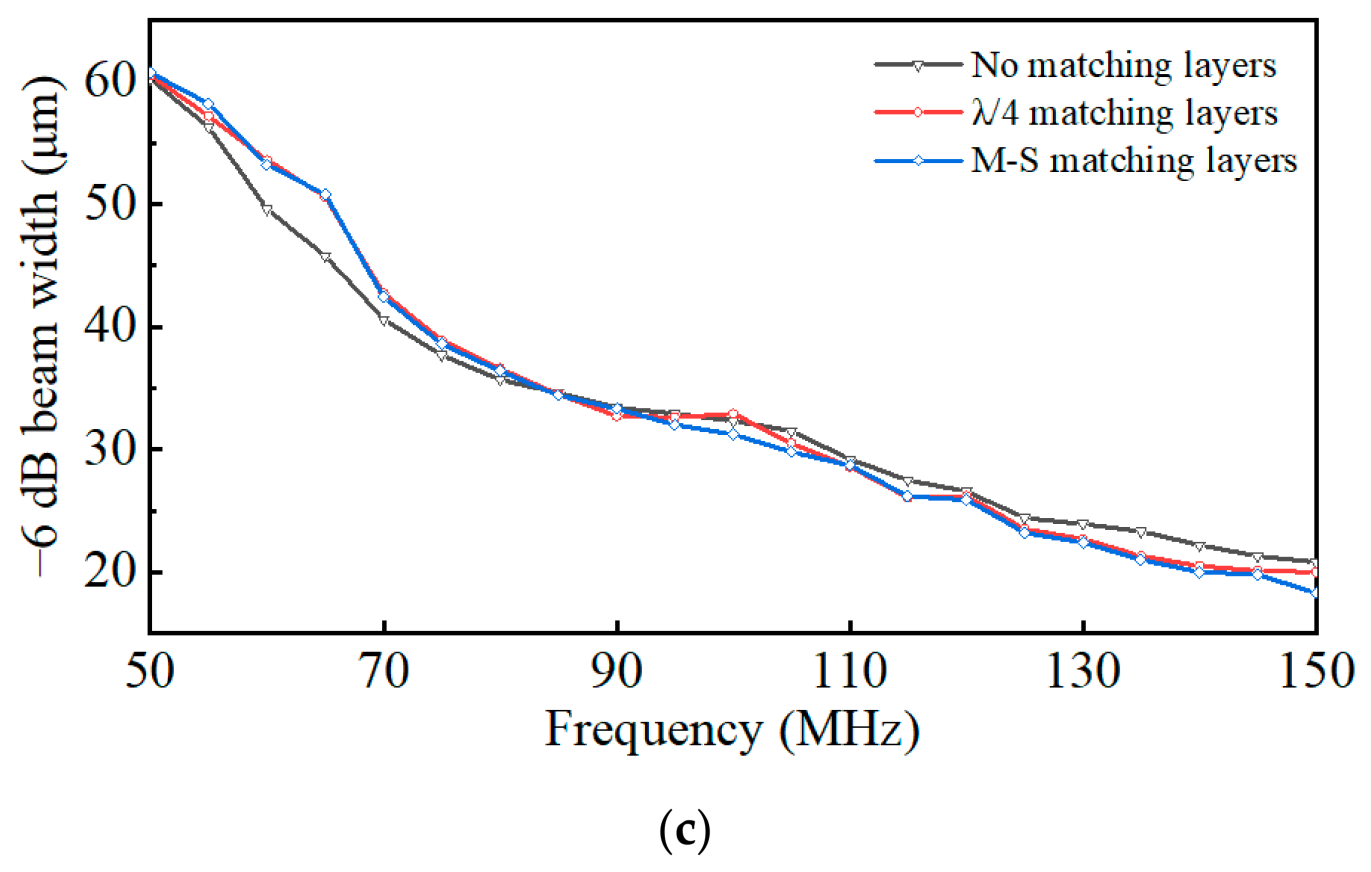
Characteristics of the transducer at different excitation frequencies: (**a**) focal length, (**b**) normalized acoustic pressure amplitude at the focal point, and (**c**) −6 dB beamwidth.

Furthermore, as shown in Figure 7a, in the lower frequency range (50–70 MHz), the focal length shows significant fluctuations. Without a matching layer, the focal length drift can reach ±0.2 mm, whereas with a matching layer, although there is a slight improvement, the fluctuations remain relatively obvious. However, when the frequency reaches 70 MHz and above, the focal length stabilizes at around 1 mm, and the fluctuation range is reduced to within ±0.1 mm. Moreover, the presence or absence of the matching layer has a negligible effect on the focal length, indicating that at high frequencies, the focusing position of the acoustic field is mainly dominated by the geometric size of the transducer and the inherent properties of the material. The role of the matching layer becomes increasingly weakened.

In terms of acoustic pressure, as shown in Figure 7b, with the presence of a matching layer, the acoustic pressure at the focal point increases overall, but the increase amplitude shows frequency dependence; near the central frequency of 100 MHz, the acoustic pressure is significantly higher compared to without a matching layer, highlighting the efficient energy transmission of the matching layer near the resonant point. At lower frequencies (50 MHz) and higher frequencies (150 MHz), the acoustic pressure increase is not obvious, indicating that the optimization effect of the matching layer is concentrated within the design bandwidth, and the performance at the band edges deteriorates due to impedance mismatch. Both the focal distance and the –6 dB beamwidth remain essentially independent of excitation frequency for the λ/4 Parylene-C case and the M-S (Au/Parylene-C) case alike—both quantities are dictated by the fixed aperture curvature (effective f-number) and diffraction scaling, not by the matching layer implementation. The focal pressure amplitude, by contrast, exhibits a pronounced frequency dependence, being governed by the frequency-varying front-face mismatch and transmission loss of each matching scheme. Compared to the λ/4 Parylene-C matching layer, the mass–spring stack delivers systematically higher focal pressures; notably, near the design frequency (~100 MHz), the acoustic pressure amplitude of the mass–spring matching layer at the focus is 1.5 times that of λ/4 Parylene-C case. These simulation results confirm the broad-band advantage of the matching layer design in the document, while also revealing its limitations, providing an important reference for the frequency optimization of high-frequency transducers.

As shown in Figure 7c, we have calculated the −6 dB beamwidth at different excitation frequencies. Since the wavelength is inversely proportional to the frequency, under the condition that the frequency remains constant, the beamwidth decreases monotonically with the increase in the excitation frequency. This relationship has been verified in our acoustic field simulation based on COMSOL Multiphysics, as under the same transducer geometric structure (fixed curvature radius and aperture), the lateral pressure profile of the focal plane extracted by scanning the excitation frequency (from 50 MHz to 150 MHz) shows that the –6 dB beamwidth significantly narrows with the increase in the frequency, and whether to add a matching layer hardly affects this result. Especially, at an excitation frequency of 100 MHz, the −6 dB beamwidth is about 31 μm.

## 4. Discussion and Conclusions

This study addresses the core contradiction between effective focusing and high acoustic transmission efficiency of ultra-high-frequency ultrasonic transducers. Through two innovative designs, it has overcome the traditional limitations. On the one hand, the half-concave piezoelectric element adopts uneven thickness to induce multi-vibration mode coupling, breaking the bottleneck of narrow bandwidth under uniform thickness design. The traditional design relies on a single vibration mode, which is prone to focus instability due to frequency sensitivity. However, multi-mode coupling enables the working bandwidth to expand from narrow band to 50–150 MHz. Simulations also verify that its acoustic focusing ability is significantly superior to the conventional structure, providing a new path for the precise positioning of tiny defects (flip-chip solder joint defects) at ultra-high frequencies. On the other hand, the new acoustic matching layer based on the mass–spring model effectively alleviates the huge impedance difference between the piezoelectric element and the detection medium (traditional matching layers often suffer a sudden drop in efficiency due to a single material property). This significantly enhances the acoustic pressure at the focus and maintains stable transmission within the broadband range. This work successfully integrates a thickness-graded hemi-concave piezoelectric element with a vacuum-deposited acoustic matching layer, bridging the methodological gap in the synergistic design of "broadband, high-efficiency, and focused" ultra-high-frequency transducers. The simulation conclusions also provide an operational direction for subsequent experimental fabrication.

By designing an ultra-high-frequency transducer based on half-concave piezoelectric elements, where the maximum center frequency is determined by the thinnest thickness of the element, we utilized non-uniform thickness to induce the coupling of multiple vibration modes, thereby expanding the working bandwidth beyond the traditional uniform thickness design. The simulation results demonstrated that this structure exhibited excellent acoustic focusing capability. Additionally, a novel acoustic matching layer based on the mass–spring model was proposed to alleviate the acoustic impedance mismatch problem. The simulation results clarify that the focal distance and the transverse –6 dB beamwidth of the half-concave LiNbO_3_ element are governed primarily by its fixed aperture geometry and the associated diffraction scaling, so these quantities change with excitation frequency yet remain essentially indifferent to whether a matching layer is introduced. A frequency sweep from 50 to 150 MHz is executed within a fully coupled COMSOL model that combines solid mechanics, electrostatics with the piezoelectric coupling, and a pressure acoustic domain for the water load, while a search tool is applied over the sweep outputs to locate the operating point where front-face mismatch is minimized, thereby identifying the effective matching condition without imposing any prescribed quarter-wavelength thickness. Under this search-based procedure, the focal plane’s lateral profiles show the expected diffraction-limited trend, with the –6 dB beamwidth narrowing as frequency rises and reaching roughly 31 μm near the design point at 100 MHz, which confirms that the performance gain demonstrated by the mass–spring stack originates from improved acoustic power transfer at the front face rather than from any alteration of the diffractive geometry itself.

The discussion further emphasizes why the mass–spring, metal–polymer route is better suited to this regime than the conventional λ/4 approach. A quarter-wavelength Parylene-C layer would demand about 6.0 μm at 100 MHz, a thickness that forces global lapping and cannot be maintained uniformly on a curved surface where local contact varies with azimuth, whereas the adopted stack—Au near 0.25 μm and Parylene-C near 2.5 μm—rests entirely within a deposition-controlled, sub-micron-to-low-micron window that conforms to the concave shape. The outcome is a focal pressure amplitude at approximately 100 MHz that is roughly 1.5 times that produced by the λ/4 Parylene-C reference, showing that ultra-high-frequency matching on curved piezoelectric elements benefits more from a numerically located, broadband-compatible matching state than from enforcing a single-frequency geometric rule. While the existing experimental literature [18] has substantiated the physical viability of mass–spring stacks on planar substrates, the present work extends this validation to curved architectures through rigorous parametric sweeps and mesh convergence studies. Although experimental verification is constrained by the current lack of ultra-high-frequency metrology infrastructure, such as calibrated micro-hydrophone systems, the current study provides a complete, reproducible, and traceable numerical framework that stands on its own as a theoretical foundation for the application of ultra-high-frequency ultrasound in micro-defect detection, and has provided insights for the broadband design of high-performance transducers and efficient energy transmission.

## Figures and Tables

**Table 1:** Material parameters for finite element simulation [18].

Materials	*v* (m/s)	*ρ* (kg/m^3^)	*Z* (MRayl)	*d* (μm)
LiNbO_3_	7360	4688	34.5	--
Au	3240	19,700	63.8	0.25
Parylene C	2350	1100	2.58	25
Epoxy resin	2650	1150	3.05	--
E-Solder	1850	3200	5.92	500
Water	1500	1000	1.5	1500

## Data Availability

The original contributions presented in this study are included in the article. Further inquiries can be directed to the corresponding author.

## Abbreviations

The following abbreviations are used in this manuscript:

M-S	Mass–spring
2D	Two-dimensional
PML	Perfectly matched layer
LiNbO_3_	Lithium niobate
